# Signatures of criticality in efficient coding networks

**DOI:** 10.1073/pnas.2302730121

**Published:** 2024-10-01

**Authors:** Shervin Safavi, Matthew Chalk, Nikos K. Logothetis, Anna Levina

**Affiliations:** ^a^Computational Neuroscience, Department of Child and Adolescent Psychiatry, Faculty of Medicine, Technische Universität Dresden, Dresden 01307, Germany; ^b^Department of Physiology of Cognitive Processes, Max Planck Institute for Biological Cybernetics, Tübingen 72076, Germany; ^c^Institut de la Vision, INSERM, CNRS, Sorbonne Université, Paris 75014, France; ^d^International Center for Primate Brain Research, Shanghai 201602, China; ^e^Department of Computer Science, University of Tübingen, Tübingen 72076, Germany; ^f^Bernstein Center for Computational Neuroscience Tübingen, Tübingen 72076, Germany

**Keywords:** criticality, efficient coding, neural dynamics, neural computation

## Abstract

The critical brain hypothesis states that the brain can benefit from operating close to a second-order phase transition. While it has been shown that several computational aspects of sensory processing (e.g., sensitivity to input) can be optimal in this regime, it is still unclear whether these computational benefits of criticality can be leveraged by neural systems performing behaviorally relevant computations. To address this question, we investigate signatures of criticality in networks optimized to perform efficient coding. We consider a spike-coding network of leaky integrate-and-fire neurons with synaptic transmission delays. Previously, it was shown that the performance of such networks varies nonmonotonically with the noise amplitude. Interestingly, we find that in the vicinity of the optimal noise level for efficient coding, the network dynamics exhibit some signatures of criticality, namely, scale-free dynamics of the spiking and the presence of crackling noise relation. Our work suggests that two influential, and previously disparate theories of neural processing optimization (efficient coding and criticality) may be intimately related.

Attempts to understand information processing in the brain have led to formulating various optimality principles. Two major paths have been explored to uncover these principles. One starts from neural networks operate close to criticality and investigate the functional advantages of this dynamics (1, 2, also see *SI Appendix*). Another one presumes that neural networks have evolved to compute optimally (3), and efficiently encode natural input as one of the best examples (4). Despite the prevalence of both approaches, connections between theories based on closeness to criticality and efficient coding hypotheses remain elusive.

To address this shortcoming, we introduce a complementary approach. Instead of tuning the network around the critical point and evaluating its statistical information processing capabilities, we optimize a network to perform a clearly defined computation and investigate whether signatures of critical dynamics emerge in the optimized network. We focus on efficient coding, as it is a well-established and functionally relevant computation, and it is accompanied by a rich spectrum of normative models (e.g., 4), and models of neural dynamics (e.g., ref. 5).

## Results

**Fig. 1.**
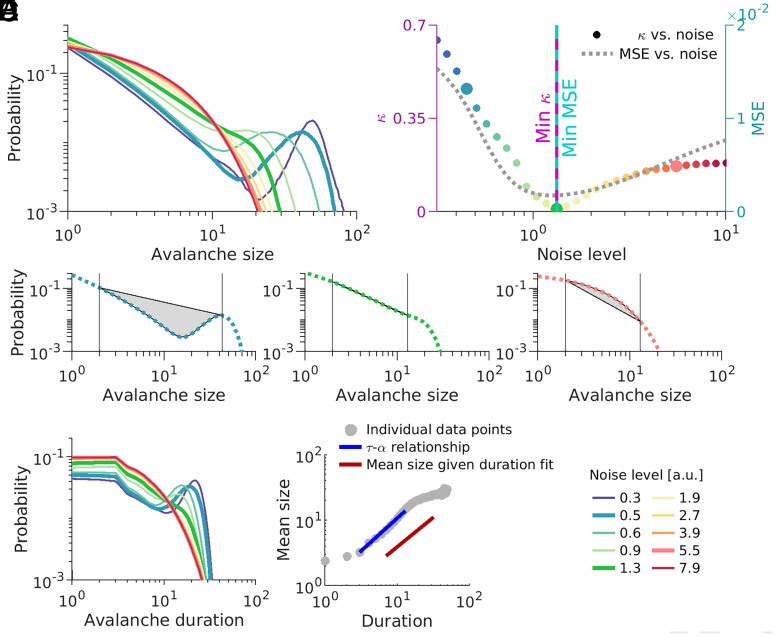
Co-occurrence of optimal points for coding and scale-freeness. (*A*) Avalanche-size distributions of efficient coding networks of size N=100 with different noise levels (indicated in the legend at the *Bottom Right*, with consistent color code across all panels). (*B*) Deviation from a power law, κ, as a function of noise level (left y-axis), color matching the panel *A*. The gray dotted line indicates a mean squared error (MSE) (right y-axis) as a function of noise. The vertical continuous line (purple) indicates the noise level corresponding to minimal κ, and the vertical broken line (cyan) indicates the noise level corresponding to minimum MSE. (*C*) Deviation from criticality measure κ for three noise levels (bold lines in *A*). *Left*: small noise (0.5, network appears supercritical); *Middle*: medium noise (1.3, network close to criticality); *Right*: strong noise (5.5, network appears subcritical). The area of gray-shaded regions between the actual avalanche size distribution and fitted power-law distribution defines the deviation measure κ. Vertical lines indicate the choices of left and right cut-offs (see main text and *SI Appendix*, for more details). Distributions for the chosen noise levels are highlighted in bold in panel *A* (matching colors). (*D*) Similar to *A*, but for avalanche durations. (*E*) Mean avalanche sizes versus durations. Each gray dot indicates a pair of duration and mean average size, and the blue line is the fitted power law. The red line indicates the predicted exponent based on the relationship between the critical exponent of avalanche sizes and durations (shifted for visualization).

We investigate signatures of criticality in a network of leaky integrate-and-fire neurons that its dynamics and connectivity are set up to optimally encode a feed-forward input (6). To assess the signatures of criticality in the efficient coding network, we investigate the distribution of neural avalanches in networks with different levels of noise. To begin, we keep the network size fixed at N=100. A neuronal avalanche is defined as an uninterrupted cascade of spikes in the network (7, also see *SI Appendix*). In a system operating close to criticality, the avalanche size (number of spikes in the cascade) follows a power-law distribution. We demonstrate that the distribution of the size of the avalanche changes systematically with the strength of the added noise (Fig. 1*A*). In networks with a small amount of noise (e.g., thick blue line in Fig. 1*A*, or Fig. 1*C*, *Left*), large avalanches dominate the distribution of avalanche sizes (a bump in the tail of the distribution signifies a transient synchronization in the network). On the other hand, for a large amount of noise (e.g., thick red line in Fig. 1*A*, or Fig. 1*C*, *Right*), the distribution is concentrated on the small avalanches (an exponential distribution). However, for intermediate levels of noise (e.g., thick green line in Fig. 1*A*, or Fig. 1*C*, *Middle*), the avalanche-size distribution resembles a power law (appears linear in the log–log coordinates), which is a key signature of criticality in neural systems (7).

To determine the most scale-free avalanche distribution, we use the measure κ (*SI Appendix*). κ is defined as the normalized area between the empirical and the ideal (fitted power law for the portion of data between two cut-offs) distribution (Fig. 1*C*). κ takes small (close to zero) values for a scale-free distribution (Fig. 1 *C*, *Middle*) and deviates from zero otherwise (Fig. 1*C*, *Left* and *Right*).

We measure how deviations from a power law, κ, and the network’s reconstruction error depends on the noise strength. We confirm the previous observation that the performance of this network depends nonmonotonically on the noise amplitude (Fig. 1*B*, gray curve), with the optimal performance achieved for an intermediate noise level (6). Interestingly, the change in κ with the noise level demonstrates a similar nonmonotonic behavior (Fig. 1*B*, colorful circles). Remarkably, both are minimized at the same noise level, resulting in a coincidence of the optimal point for coding and the most scale-free distribution (Fig. 1*B*).

**Fig. 2.**
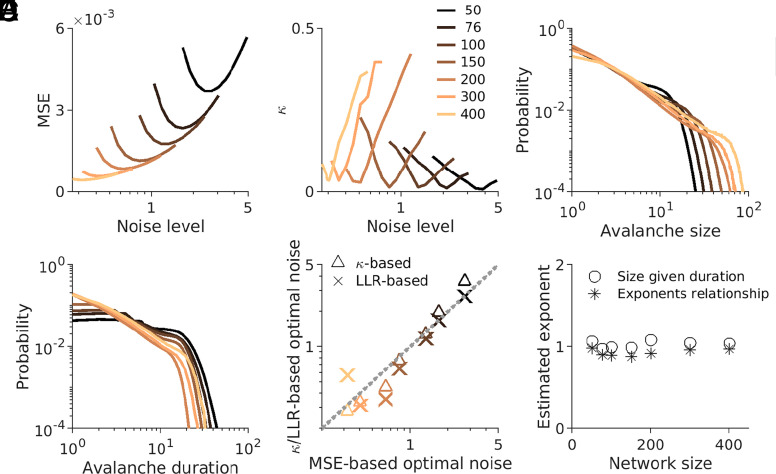
Analysis across networks of different sizes. (*A*) Mean square error (MSE) of stimulus reconstruction for different injected noise amplitudes (similar to Fig. 1*B*, gray line). Curves with different colors correspond to different network sizes (specified in the legend of panel *B*). (*B*) Deviation measure (κ) as a function of noise (similar to Fig. 1*B*, colorful dots). (*C* and *D*) Avalanche size (*C*) and duration (*D*) distributions for the noise levels corresponding to the smallest kappa. Avalanche distributions with different colors correspond to different network sizes (legend of panel *B*). (*E*) Optimal noise based on the distance to the power law (minimum of κ or the largest LLR) versus optimal noise for coding (minimum MSE). (*F*) Estimated exponents of avalanche size scaling with avalanche duration (∘) and the predicted exponent (∗) from the relationship between exponents of avalanche size (τ) and duration distributions (α) based on $\frac{\alpha -1}{\tau -1}=\frac{1}{\sigma \nu z}$ (for more details, see *SI Appendix*). The closeness of these exponents for different network sizes corresponds to a theoretically predicted relationship. For all the networks, exponents are calculated at the noise levels corresponding to the smallest κ.

We observe similar behavior for the distribution of avalanche duration (Fig. 1*D*), although the network is driven by a strong input (also see *SI Appendix*, for more details). We further analyzed the power-law exponents for the noise level with the most scale-free distribution based on κ (Fig. 2*E* and *SI Appendix*). We found that at the optimal noise level, the scaling exponent results from the mean size given duration distribution (Fig. 1*E*), matches well with the predicted scaling exponent by the relationship between the critical exponent of avalanche size and duration (Fig. 1*E*, red line; and see ref. 8). Overall, these results offer additional support to the criticality hypothesis of the brain, namely that the various information processing measures are optimized close to the critical point (2).

Next, we verify the stability of this result in terms of changes in the network’s size by considering networks of various sizes (50 to 400 neurons). We find that all networks demonstrate similar nonmonotonic behavior for the dependence of reconstruction error (Fig. 2*A*) and the scale-freeness deviation measure (Fig. 2*B*) on the strength of the noise. As expected, this nonmonotonic behavior of the reconstruction error is less pronounced for larger networks (*SI Appendix*). Furthermore, the location of the cut-off of the scale-free distribution shifts right (to the larger values) with the size of the network (Fig. 2*C*), hinting at the correct tendency in the finite-size scaling behavior (see, e.g., refs. 7 and 9) across different network sizes. Avalanche durations also follow scale-free distribution for the same noise levels (Fig. 2*D*). However, the temporal dynamics of the network are heavily affected by the constraints imposed by design principles of our efficient coding network (see *SI Appendix* for more details).

We observe the co-occurrence of efficient coding optimality with criticality optimality across all network sizes. The noise levels where coding error is minimal (x-coordinates in Fig. 2*E*) and where κ is minimal (y-coordinates in Fig. 2*E*) are highly correlated across different network sizes. This observation is also robust to variations in the choice of the right cut-off needed for calculating κ. Furthermore, our κ-based analysis is compatible with the assessment based on the log-likelihood ratio (LLR) of power-law to log-normal and exponential distributions (see Fig. 2*E*, star symbols, and *SI Appendix*). Lastly, we computed the scaling exponent relationships, as has been suggested to be one of the most reliable signatures of criticality (see, e.g., refs. 2, 8, 10 and *SI Appendix*). The scaling relationship is predicted based on size and duration critical exponents (Fig. 2*F*, stars) and measured based on fitting a power law to the mean size given the duration data (Fig. 2*F*, circles) are in agreement with each other across all network sizes. Thus, our analysis suggests that the estimated scaling exponents are stable across network sizes and consistent across methods Fig. 2*F*), which further supports our conclusion.

## Discussion

In this study, we probe the connection between the optimality discussed in the context of the criticality hypothesis of the brain and the optimality discussed in theories of neural computations. To this end, we examine an efficient coding networks (6, 11) for signatures of criticality. We find signatures of criticality (scale-free dynamics of the neural avalanches and crackling noise relation) emerging in a network that was designed based on criticality-agnostic principles merely by optimizing the coding performance. This suggests criticality and efficient coding are intimately related.

Our approach contrasts with previous studies investigating the criticality hypothesis, which used models (e.g., a branching network) that can attain various (critical/noncritical) states depending on a limited number of control parameters (e.g., branching ratio) and then quantified how the computational primitives (2), such as sensitivity to input, depend on these control parameters. These models aim at reproducing realistic neuronal dynamics. They are typically driven by a slowly delivered noise and have no specific input and no read-out strategy. Therefore, such studies are largely agnostic to computational objectives central to theories of neural computation. Our approach thus opens the door to new questions about the relevance of criticality for precisely defined and task-relevant computations.

Future research should address why and how critical dynamics enables optimal efficient coding. For instance, it is unclear what the exact role of neural avalanches is or why and how their scale-free distribution may be optimal for neural coding. Answering such questions requires going beyond our simulation-based approach. For instance, similar to Timcheck et al. (12), mathematical analysis is needed to understand how the distribution of avalanches depends on different attributes of the network (noise, delay, connection weights, etc.). Furthermore, we need to establish how those attributes affect the coding optimality. In particular, the presence of neuronal synchronization in our network (due to synaptic delay) resonates well with recent developments in the field of neural criticality (see, e.g., ref. 13), which might also be insightful for understanding underlying mechanisms.

Overall, there are several approaches to gaining a mechanistic insight for our observations of criticality and efficient coding optimality co-occurrence. Having this mechanistic understanding will also allow us to extend this framework to more sophisticated computations. We believe that our study opens promising avenues for future investigations to establish the connection between other aspects of criticality (2) and theories of neural computations (3).

## Materials and Methods

We used the network of ref. 6 and tuned it for efficient coding (e.g., via a stochastic gradient descent algorithm); and evaluated the signatures of criticality in the spiking activity. Further details are provided in *SI Appendix*.

## Supplementary Material

Appendix 01 (PDF)

## Data Availability

Codes have been deposited in GitHub (https://github.com/shervinsafavi/Safavi_etal_PNAS_2024) (14).

## References

[1] M. A. Muñoz, Colloquium: Criticality and dynamical scaling in living systems. Rev. Mod. Phys. **90**, 031001 (2018).

[2] J. M. Beggs, The Cortex and the Critical Point: Understanding the Power of Emergence (MIT Press, Cambridge, MA, 2022).

[3] P. Dayan, L. F. Abbott, *Theoretical Neuroscience: Computational and Mathematical Modeling of Neural Systems. Computational Neuroscience* (MIT Press, 2001).

[4] M. Chalk, O. Marre, G. Tkačik, Toward a unified theory of efficient, predictive, and sparse coding. Proc. Natl. Acad. Sci. U.S.A. **115**, 186–191 (2018). 29259111 10.1073/pnas.1711114115 PMC5776796

[5] J. Kadmon, J. Timcheck, S. Ganguli, Predictive coding in balanced neural networks with noise, chaos and delays. Adv. Neural Inf. Process. Syst. **33**, 16677–16688 (2020).

[6] M. Chalk, B. Gutkin, S. Deneve, Neural oscillations as a signature of efficient coding in the presence of synaptic delays. eLife **5**, e13824 (2016). 27383272 10.7554/eLife.13824 PMC4959845

[7] J. M. Beggs, D. Plenz, Neuronal avalanches in neocortical circuits. J. Neurosci. **23**, 11167–11177 (2003). 14657176 10.1523/JNEUROSCI.23-35-11167.2003 PMC6741045

[8] N. Friedman , Universal critical dynamics in high resolution neuronal avalanche data. Phys. Rev. Lett. **108**, 208102 (2012). 23003192 10.1103/PhysRevLett.108.208102

[9] A. Levina, J. M. Herrmann, T. Geisel, Dynamical synapses causing self-organized criticality in neural networks. Nat. Phys. **3**, 857–860 (2007).

[10] M. A. Muñoz, R. Dickman, A. Vespignani, S. Zapperi, Avalanche and spreading exponents in systems with absorbing states. Phys. Rev. E **59**, 6175–6179 (1999). 10.1103/physreve.59.6175 11969602

[11] M. Boerlin, C. K. Machens, S. Deneve, Predictive coding of dynamical variables in balanced spiking networks. PLoS Comput. Biol. **9**, e1003258 (2013). 24244113 10.1371/journal.pcbi.1003258 PMC3828152

[12] J. Timcheck, J. Kadmon, K. Boahen, S. Ganguli, Optimal noise level for coding with tightly balanced networks of spiking neurons in the presence of transmission delays. PLoS Comput. Biol. **18**, e1010593 (2022). 36251693 10.1371/journal.pcbi.1010593 PMC9576105

[13] S. di Santo, P. Villegas, R. Burioni, M. A. Muñoz, Landau-Ginzburg theory of cortex dynamics: Scale-free avalanches emerge at the edge of synchronization. Proc. Natl. Acad. Sci. U.S.A. **115**, E1356–E1365 (2018). 29378970 10.1073/pnas.1712989115 PMC5816155

[14] S. Safavi, shervinsafavi/Safavi_etal_PNAS_2024. GitHub. https://github.com/shervinsafavi/Safavi_etal_PNAS_2024. Deposited 19 September 2024.

