# Supplementary material for: Signatures of criticality in efficient coding networks
Source: Proc Natl Acad Sci U S A. 2024 Oct 1;121(41):e2302730121. doi: 10.1073/pnas.2302730121 (PMC11474077; doi:10.1073/pnas.2302730121)
Supplement: Supplementary file 1 — Appendix 01 (PDF) [file pnas.2302730121.sapp.pdf]

Supporting Information for

Signatures of criticality in efficient coding networks

Shervin Safavi, Matthew Chalk, Nikos Logothetis, Anna Levina

Corresponding Author name.
E-mail: research@shervinsafavi.org, anna.levina@uni-tuebingen.de, matthew.chalk@inserm.fr

This PDF file includes:

Supporting text
SI References

Supporting Information Text

Materials and methods

Efficient coding network. The neuronal network model used in this study was introduced and described extensively in the previous studies (1, 2), thus we restrict ourselves to a brief explanation of the key aspect of the model. Our network can be optimized to encode a sensory input efficiently (i. e., with a minimal number of spikes) and accurately (i. e., with minimal reconstruction error). The network optimization objective is incorporated in the loss function $E(t)$,

$$E(t) = (x(t) - \widehat{x}(t))^2 + \alpha \sum_i r_i(t) + \beta \sum_i r_i(t)^2 \,, \qquad [1]$$

where $x(t)$ is a given one-dimensional sensory input (similar to 2, 3), $\widehat{x}(t)$ is the reconstructed sensory input, $r_i(t)$ is the firing rate of the neuron i, and α and β are the weights of the $L1$ and $L2$ penalties on the firing rate.

It is assumed that the input can be reconstructed by performing a linear readout of the spike trains, more precisely, by a weighted leaky integration of output spike trains,

$$\tau \frac{d\hat{x}(t)}{dt} = -\hat{x}(t) + \sum_i w_i o_i(t) \,, \qquad [2]$$

where o_i indicates the output spike trains for the neuron i,

$$o_i(t) = \sum_k \delta\left(t - t_i^k\right) \,, \qquad [3]$$

and τ is the read-out time constant*, and w_i is a constant read-out weight associated to the neuron i.

Given an idealized network with instantaneous synapses, the optimal network could be derived from the first principles. Boerlin *et al.* (1) demonstrated that the dynamics of each leaky-integrate and fire (LIF) neuron can be expressed by conventional differential equation governing the dynamics of the membrane potentials,

$$\tau \frac{dV_i(t)}{dt} = -V_i(t) + w_i c(t) - w_i \sum_k w_k o_k(t) - \beta o_i(t) + \sigma \nu_i(t) \,, \qquad [4]$$

where V_i is the membrane potential of the neuron i, w_i is the constant readout which was introduced in Equation 2, $c(t)$ is the input to the network, $o_i(t)$ is the spike train of neuron i, β is the regularizer that was introduced in Equation 1, and $\nu(t)$ is a white noise with a unit variance that was manually added in the original derivation of (1) for biological realism. Notably, in this network, we have two types of input: a feed-forward input, $w_i c(t)$ and a recurrent input $-w_i \sum_k w_k o_k(t)$. The recurrent input is the result of a fully connected network. In this network, neurons that receive a common input decorrelate their activity to avoid communicating redundant information via instantaneous recurrent inhibition.

Chalk *et al.* (2) introduced a more biologically plausible variant of (1) 's network by incorporating synaptic delays and introducing a balanced network of inhibitory and excitatory population of neurons. They incorporated realistic synaptic delays by assuming that each spike generates a continuous current input to other neurons, with a dynamic that is described by the conventional alpha function,

$$h(t) = \begin{cases} \frac{1}{\tau_d - \tau_r}\left[e^{\frac{-(t-\tau_{tr})}{\tau_d}} - e^{\frac{-(t-\tau_{tr})}{\tau_r}}\right] & \text{if } t > \tau_{tr} \\ 0 & \text{if } t < \tau_{tr} \,, \end{cases} \qquad [5]$$

where τ_r and τ_d are respectively synaptic rise and decay times. Adding realistic synaptic delays led to network synchronization, which impairs coding efficiency. Chalk *et al.* (2) demonstrated that, in the presence of synaptic delays, this network of LIF neurons can nonetheless be optimized for efficient coding by adding noise to the network. In this study, we implement the additional noise, as white noise added to the membrane potentials. However, (2) also demonstrated similar dependency of the network's performance to noise by using other ways of incorporating noise, for instance, by inducing unreliability in spike elicitation (3) or tuning the L^1 or L^2 norm (4, 5) which controls the spiking threshold (also see, 6, for other approaches).

The original network introduced by (1) was a pure inhibitory network. Chalk *et al.* (2) introduced a variant of this network that respects Dale's law (and used in this study). In their network, they introduce a population of inhibitory neurons that tracks the estimate encoded by the excitatory neurons, and provides recurrent feedback to the excitatory population (for further detail see, 1, 2). Also see (7) for other approaches.

We find that all networks demonstrate a non-monotonic behavior for the dependence of reconstruction error on the strength of the noise (Figure 2A in the main text). However, this non-monotonic behavior of the reconstruction error is less pronounced for larger networks. This is expected because the recurrent network used in our study is particularly suitable to code a single dimension of input by a small number of neurons (1, 2), i. e., around hundred, rather than thousands, of neurons per input dimension (see 5, as to how this problem could be alleviated for large networks by encoding higher dimensional inputs).

* In the efficient coding network used in this study (as in 2), for simplicity, the readout time constant of the input (i.e., the timescale of $x(t)$) is the same as the time constant of the membrane potential of the neurons. Nevertheless, in (1) they are not necessarily the same for more general computations.

Brain criticality hypothesis. The theory of critical phase transition in complex systems has been successful in explaining a wide range of phenomena in nature (8, 9), and "brain criticality hypothesis" (10–13), has been developed based on this solid foundation. In a nutshell, the brain criticality hypothesis states that, the brain operates close to a critical state, a state on the edge of transition between order and disorder. The first experimental evidence on scale-freeness of the brain dynamics (as one of the signatures of criticality) has been reported almost two decades ago (14). Later on, such scale-free dynamics has also been observed in various other (smaller and larger) scales; for instance, at the scale of actin in dendrites (15), at the scale of neuronal membranes (16), at the scale of the entire brain (17). Moreover, being close to this state is beneficial for the brain as an information processing system (see, 18–21, for a reviews), as it has been shown that several information processing capabilities, such as sensitivity to input (22, 23), dynamic range (22, 24, 25), or information transmission and storage (26–29), and various other computational characteristics have also been considered to be relevant (30–41).

Various empirical and theoretical investigations lend support to the brain criticality hypothesis, and signify the potential functional relevance of the brain criticality hypothesis. Therefore, it has been motivating to search for diverse signatures of criticality in the brain. These signatures can be categorized into three groups: scale-freeness neural activity (avalanche criticality), the dynamical regime of the neural system (edge of bifurcation criticality), and thermodynamics of the neural data (maximum entropy criticality). Here, we focused on scale-free distribution of neural avalanches as one of the criticality signatures, however, all signatures are briefly introduced in the following.

Scale-freeness neural activity (avalanche criticality): Scale-free cascade of activity is a ubiquitous type of dynamics in nature: For instance, in interacting tectonic plates (42), forest fires (43), nuclear chain reactions (44), and because the units of the system are coupled to each other, similar threshold-crossing events *propagate* through other units of the system. Such propagating dynamics can lead to large *avalanches* of activity. Almost two decades ago, Beggs and Plentz (14) observed similar cascades in the activity of in-vitro neural populations and later on, others reported such scale-free cascades at various other neuronal recordings in various scales (see references in, 12, 45). It has been suggested critical systems not only should show the mentioned scale-free dynamics, but also they should follow the scaling laws (46), that were observed in neural data (47) as well.

The dynamical regime of the neural system (edge of bifurcation criticality): When a dynamical system has a transition from one dynamical regime to another (such as a transition from order to chaos), it experiences a *bifurcation* (48–50). The point where the transition happens (in certain conditions) is also denoted as the critical point. There are various kinds of bifurcations (see, 48), but some of them have been particularly appealing for understating the dynamics of the brain as well as computation in the brain. Briefly, transitioning from order to chaos (51), and transitioning from an asynchronous to a synchronous state (52) have been considered as two important bifurcations for the brain (for further elaboration see, 12, 50, and references therein). Along the same line, other empirical signatures have also been investigated recently; for instance, Safari *et al.* (53) employ spike-field coupling (54, 55) as an indicator of operating close to criticality. Lastly, avalanche criticality and bifurcation criticality can co-occur, when there is a continuous phase transition (50) (for example, see, 56, 57), nevertheless, it has been suggested that these two types of criticality do not necessarily co-occur (58) and, therefore, should be attributed to two distinct phenomena.

Thermodynamic of the neural data (maximum entropy criticality): Statistical mechanics provides a powerful framework to study collective behavior in systems consisting of interacting units with many degrees of freedom (59). Tools from statistical mechanics have been applied in neural networks in order to understand their collective dynamics (60). Along the same line, for instance, Tkačik *et al.* (61) approached the activity of neurons from a thermodynamical perspective. They define a Boltzman-like distribution, derive various thermodynamic quantities, such as heat capacity, based on the estimated Boltzman distribution, and ultimately define criticality based on thermodynamic quantities (like divergence of heat capacity). Moreover, in empirical data, this novel framework is applicable and functionally relevant. This novel formulation introduces another signature or definition of criticality in neural system (61).

Avalanche detection. To investigate the scale-free characteristic of the spiking activity (as a potential signature of networks operating close to criticality, 12, 13), similar to previous studies (e.g., 14), we probe the distribution of neural avalanches. A neuronal avalanche is defined as an uninterrupted cascade of spikes in the network (14). In a system operating close to criticality, the distribution of avalanche sizes (number of spikes in a cascade) and avalanche lifetime follows a power law.

For detecting the avalanches, we followed the procedure used in previous studies (e. g., 14). The period of no spiking activity signifies the end of the previous avalanche if it is larger than a threshold Δ. We used the mean inter-spike interval ($\Delta = \langle ISI \rangle$) in the compound spike train (obtained by collapsing the spike trains of all neurons onto a single timeline) as a threshold. Thus, when the compound spike train is interrupted for an interval greater than Δ, we consider that the current avalanche is over, and the next spike will be the onset of a new avalanche. The size of the avalanche is the number of spikes between these two silent time points. A slightly different procedure has also been used for avalanche detection. In the alternative approach, for computing the Δ, one counts the synchronous spike only once, i. e., excluding zero ISIs. Notably, similar results were obtained using the alternative method.

This choice of threshold Δ potentially can be made separately for individual networks with different noise levels. However, to avoid introducing additional variability across different levels of noise, we fixed the threshold for all the noise levels. For a given network size, we took the threshold from the network with the noise level corresponding to minimal mean square error (MSE), and use that for all noise levels. We also checked that taking slightly different thresholds would not change the results of our study.

Closeness to criticality assessment. We consider the scale-free distribution of neural avalanche as a signature of criticality in the network (62, 63). Thus, to determine the most scale-free avalanche distribution, we introduce a deviation measure κ, which quantifies the deviation from an ideal power-law distribution. Our κ measure closely follows the non-parametric measure introduced by Shew *et al.* (64), but does not assume a particular scaling exponent, which might be important because we do not know *a priori* what is the relevant universality class for neuronal avalanches (see, e. g., 65). We define κ as the area between the empirical and the ideal (fitted power-law) distribution, normalized by the number of data points (in the empirical distribution) between left and right cut-offs. Larger values of κ correspond to larger deviations from the power-law distribution. Power laws were fitted between two cut-offs. The left cut-off was always chosen to be 2 (i. e., avalanches with at least two spikes). The right cut-off is typically chosen subjectively based on the problem at hand (66), here, we swept over a wide range of choices to be as objective as possible. We choose it between two possible options: it was either a certain percentile of the number of avalanches (within the range of 50-95%), or a fraction of the network size (within the range of 10-25%). Between the mentioned choices above, the one that led to the inclusion of more data, i. e., a larger proportion of avalanches, was selected The ideal power-law distribution was also determined based on a linear fit between the final choices of left and right cut-offs. Notably, the results were robust to variations (in the ranges noted above) in the choice of cut-offs (see Figure 2C of the main text).

Temporal dynamics of the network. The constraints imposed by the design principles of our efficient coding network heavily affect the network's temporal dynamics. First, the network in our study is driven by a strong external input which the network encodes. Thus, in general settings, the temporal dynamics of the network is severely affected by the temporal dynamics of the input (as the whole purpose of the network is *encoding the input*). Second, as shown in previous studies (2), the network elicits characteristic bursts of spikes as a correction mechanism that severely deforms the shape of avalanches. Lastly, the network is explicitly discouraged from generating long cascades of spikes. Firing more spikes in our networks means a larger encoding error. Thus, the network elicits a very strong inhibition to correct for the encoding error. This indicates another factor that heavily affects the temporal dynamic of the network. It is particularly important to consider this when investigating the scale-freeness of avalanche duration. As the network is discouraged from generating long spiking cascades, there will be fewer long-lasting avalanches, which is essential for statistical analysis of heavy-tail distribution, particularly power laws.

Statistical analysis of avalanches and exponents. For assessing the goodness of power-law fit to neural avalanches, similar to recent studies (e.g., 67–69), we use the likelihood ratio (LLR) of power-law to other distributions. We use the `powerlaw` package developed by (66) that was established based on methodology introduced by (70). We computed the LLR between a power-law and a log-normal (that is, the strongest competitor model) and an exponential distribution for a given collection of avalanche sizes. In this statistical analysis, we use the significance threshold of 0.05 for both of the comparisons. For this analysis, two cut-offs (lower and upper bounds, x_{min} and x_{max} respectively) also needed to be set. The lower bound, x_{min}, was chosen automatically based on the procedure described in (70) which is implemented in `powerlaw` package of (66), and for the upper bound, x_{max}, we use the x_{max} corresponds to 95% percentile of number of avalanches (to include a large portion of avalanches).

We computed scaling exponents relationships (see e.g., 47, 71, and our results, Figure 2F in the main text),

$$f(S) \sim S^{-\tau}\,, \qquad [6]$$

$$f(T) \sim T^{-\alpha}\,, \qquad [7]$$

$$\frac{\alpha - 1}{\tau - 1} = \frac{1}{\sigma \nu z}\,, \qquad [8]$$

both scaling relationships of critical exponents (Equation 8), and the scaling exponent of mean size versus duration (Equation 9),

$$\langle S \rangle (T) \sim T^{1/\sigma \nu z}\,, \qquad [9]$$

as it has been suggested (47, 65, 71, 72) to be one of the most reliable signatures of criticality among others (but also see, 73–75). We use different approaches to compute the relationship between the size and duration of the avalanches. In our network, avalanches occur between two large bursts (first originated from the activity overshoot due to the delay, and the second – the inhibitory compensation that stops the activity for a while). To compute the avalanche shape, we remove these bursty edges of the avalanches by cutting one bin from the start and the end of the avalanche. After that, the avalanche shape resembles the conventionally expected bell-shape. We used the NCC toolbox (76) to compute the exponent relationships based on size given duration and shape collapse (all at the noise level corresponds to the smallest κ). For the analysis of avalanche shapes, we also followed the guideline of (76) and removed avalanches with a duration smaller than a cutoff from the left (6, 4, 3, 2, 2, 2, and 2, respectively for network of size 50, 76, 100, 150, 200, 300 and 400) and larger than a cutoff from the right (14, 17, 13, 13, 13, 16, and 19 respectively for network of size 50, 76, 100, 150, 200, 300 and 400).

References

1. M Boerlin, CK Machens, S Deneve, Predictive coding of dynamical variables in balanced spiking networks. *PLoS computational biology* **9**, e1003258 (2013).

2. M Chalk, B Gutkin, S Deneve, Neural oscillations as a signature of efficient coding in the presence of synaptic delays. *eLife* **5** (2016).
3. J Timcheck, J Kadmon, K Boahen, S Ganguli, Optimal noise level for coding with tightly balanced networks of spiking neurons in the presence of transmission delays. *PLOS Comput. Biol.* **18**, e1010593 (2022).
4. V Koren, S Deneve, Computational Account of Spontaneous Activity as a Signature of Predictive Coding. *PLoS computational biology* **13**, e1005355 (2017).
5. N Calaim, FA Dehmelt, PJ Gonçalves, CK Machens, The geometry of robustness in spiking neural networks. *eLife* **11**, e73276 (2022).
6. CER Buxó, JW Pillow, Poisson balanced spiking networks. *PLOS Comput. Biol.* **16**, e1008261 (2020).
7. V Koren, S Panzeri, Biologically plausible solutions for spiking networks with efficient coding in *Advances in Neural Information Processing Systems.* (2022).
8. C Mathis, T Bhattacharya, SI Walker, The Emergence of Life as a First-Order Phase Transition. *Astrobiology* **17**, 266–276 (2017).
9. DR Chialvo, Life at the edge: Complexity and criticality in biological function. *ArXiv181011737 Q-Bio* (2018).
10. T Mora, W Bialek, Are Biological Systems Poised at Criticality? *J Stat Phys* **144**, 268–302 (2011).
11. G Tkacik, W Bialek, Information Processing in Living Systems. *Annu. Rev Conden Ma P* **7**, 89–117 (2016).
12. MA Muñoz, Colloquium: Criticality and dynamical scaling in living systems. *Rev. Mod. Phys.* **90**, 031001 (2018).
13. J O'Byrne, K Jerbi, How critical is brain criticality? *Trends Neurosci.* (2022).
14. JM Beggs, D Plenz, Neuronal avalanches in neocortical circuits. *The J. neuroscience : official journal Soc. for Neurosci.* **23**, 11167–77 (2003).
15. M Bonilla-Quintana, F Wörgötter, E D'Este, C Tetzlaff, M Fauth, Actin in Dendritic Spines Self-Organizes into a Critical State. *bioRxiv* p. 2020.04.22.054577 (2020).
16. JK Johnson, NC Wright, J Xia, R Wessel, Single-cell membrane potential fluctuations evince network scale-freeness and quasicriticality. *J. Neurosci.* pp. 3163–18 (2019).
17. TF Varley, O Sporns, A Puce, J Beggs, Differential Effects of Propofol and Ketamine on Critical Brain Dynamics. *bioRxiv* p. 2020.03.27.012070 (2020).
18. JM Beggs, The criticality hypothesis: How local cortical networks might optimize information processing. *Philos T R Soc A* **366**, 329–343 (2008).
19. WL Shew, D Plenz, The functional benefits of criticality in the cortex. *The Neurosci. : a review journal bringing neurobiology, neurology psychiatry* **19**, 88–100 (2013).
20. N Tomen, Ph.D. thesis (2019).
21. R Zeraati, V Priesemann, A Levina, Self-Organization Toward Criticality by Synaptic Plasticity. *Front. Phys.* **9**, 103 (2021).
22. O Kinouchi, M Copelli, Optimal dynamical range of excitable networks at criticality. *Nat Phys* **2**, 348–352 (2006).
23. L Brochini, et al., Phase transitions and self-organized criticality in networks of stochastic spiking neurons. *Sci. Rep.* **6**, 35831 (2016).
24. DB Larremore, WL Shew, JG Restrepo, Predicting Criticality and Dynamic Range in Complex Networks: Effects of Topology. *Phys. Rev. Lett.* **106**, 058101 (2011).
25. T Nur, SH Gautam, JA Stenken, WL Shew, Probing spatial inhomogeneity of cholinergic changes in cortical state in rat. *Sci. Rep.* **9**, 9387 (2019).
26. WL Shew, H Yang, S Yu, R Roy, D Plenz, Information capacity and transmission are maximized in balanced cortical networks with neuronal avalanches. *The J. neuroscience : official journal Soc. for Neurosci.* **31**, 55–63 (2011).
27. F Vanni, M Lukovic, P Grigolini, Criticality and transmission of information in a swarm of cooperative units. *Phys. review letters* **107**, 078103 (2011).
28. M Lukovic, F Vanni, A Svenkeson, P Grigolini, Transmission of information at criticality. *Phys. A* **416**, 430–438 (2014).
29. D Marinazzo, et al., Information transfer and criticality in the Ising model on the human connectome. *PloS one* **9**, e93616 (2014).
30. AM Turing, I.—Computing Machinery and Intelligence. *Mind* **LIX**, 433–460 (1950).
31. T Tanaka, T Kaneko, T Aoyagi, Recurrent Infomax Generates Cell Assemblies, Neuronal Avalanches, and Simple Cell-Like Selectivity. *Neural Comput.* **21**, 1038–1067 (2008).
32. J Hidalgo, et al., Information-based fitness and the emergence of criticality in living systems. *Proc. Natl. Acad. Sci. United States Am.* **111**, 10095–100 (2014).
33. J Hidalgo, J Grilli, S Suweis, A Maritan, MA Muñoz, Cooperation, competition and the emergence of criticality in communities of adaptive systems. *J. Stat. Mech.* **2016**, 033203 (2016).
34. PAM Mediano, JC Farah, M Shanahan, Integrated Information and Metastability in Systems of Coupled Oscillators. *ArXiv160608313 Q-Bio* (2016).
35. S Khajehabdollahi, P Abeyasinghe, A Owen, A Soddu, The emergence of integrated information, complexity, and consciousness at criticality. *bioRxiv* p. 521567 (2019).
36. H Hoffmann, DW Payton, Optimization by Self-Organized Criticality. *Sci. Rep.* **8**, 2358 (2018).
37. L Michiels van Kessenich, D Berger, L de Arcangelis, HJ Herrmann, Pattern recognition with neuronal avalanche dynamics. *Phys. Rev. E* **99**, 010302 (2019).

38. R Wang, et al., Hierarchical Connectome Modes and Critical State Jointly Maximize Human Brain Functional Diversity. *Phys. Rev. Lett.* **123**, 038301 (2019).
39. K Finlinson, WL Shew, DB Larremore, JG Restrepo, Optimal control of excitable systems near criticality. *Phys. Rev. Res.* **2**, 033450 (2020).
40. R Zeraati, et al., Intrinsic timescales in the visual cortex change with selective attention and reflect spatial connectivity. *Nat Commun* **14**, 1858 (2023).
41. F Habibollahi, BJ Kagan, AN Burkitt, C French, Critical dynamics arise during structured information presentation within embodied in vitro neuronal networks. *Nat. Commun.* **14**, 5287 (2023).
42. B Gutenberg, CF Richter, Earthquake magnitude, intensity, energy, and acceleration(Second paper). *Bull. Seismol. Soc. Am.* **46**, 105–145 (1956).
43. BD Malamud, G Morein, DL Turcotte, Forest Fires: An Example of Self-Organized Critical Behavior. *Science* **281**, 1840–1842 (1998).
44. TE Harris, *The Theory of Branching Processes*, Grundlehren Der Mathematischen Wissenschaften. (Springer-Verlag, Berlin Heidelberg), (1963).
45. V Agrawal, S Chakraborty, T Knöpfel, WL Shew, Scale-Change Symmetry in the Rules Governing Neural Systems. *iScience* **12**, 121–131 (2019).
46. JP Sethna, KA Dahmen, CR Myers, Crackling noise. *Nature* **410**, 242–50 (2001).
47. N Friedman, et al., Universal critical dynamics in high resolution neuronal avalanche data. *Phys. review letters* **108**, 208102 (2012).
48. EM Izhikevich, *Dynamical Systems in Neuroscience: The Geometry of Excitability and Bursting (Computational Neuroscience).* (The MIT Press, Cambrige, Massachusetts, USA), (2010).
49. M Breakspear, Dynamic models of large-scale brain activity. *Nat. neuroscience* **20**, 340–352 (2017).
50. L Cocchi, LL Gollo, A Zalesky, M Breakspear, Criticality in the brain: A synthesis of neurobiology, models and cognition. *Prog. Neurobiol.* **158**, 132–152 (2017).
51. N Bertschinger, T Natschlager, Real-time computation at the edge of chaos in recurrent neural networks. *Neural computation* **16**, 1413–1436 (2004).
52. S di Santo, P Villegas, R Burioni, MA Muñoz, Landau–Ginzburg theory of cortex dynamics: Scale-free avalanches emerge at the edge of synchronization. *PNAS* p. 201712989 (2018).
53. N Safari, F Shahbazi, M Dehghani-Habibabadi, M Esghaei, M Zare, Spike-phase coupling as an order parameter in a leaky integrate-and-fire model. *Phys. Rev. E* **102**, 052202 (2020).
54. S Safavi, NK Logothetis, M Besserve, From Univariate to Multivariate Coupling between Continuous Signals and Point Processes: A Mathematical Framework. *Neural Comput.* pp. 1–67 (2021).
55. S Safavi, et al., Uncovering the organization of neural circuits with Generalized Phase Locking Analysis. *PLOS Comput. Biol.* **19**, e1010983 (2023).
56. MO Magnasco, O Piro, GA Cecchi, Self-tuned critical anti-Hebbian networks. *Phys. review letters* **102**, 258102 (2009).
57. F Pittorino, M Ibáñez-Berganza, M di Volo, A Vezzani, R Burioni, Chaos and Correlated Avalanches in Excitatory Neural Networks with Synaptic Plasticity. *Phys. Rev. Lett.* **118**, 098102 (2017).
58. K Kanders, T Lorimer, R Stoop, Avalanche and edge-of-chaos criticality do not necessarily co-occur in neural networks. *Chaos* **27**, 047408 (2017).
59. J Sethna, LoAaSSPJP Sethna, *Statistical Mechanics: Entropy, Order Parameters, and Complexity.* (OUP Oxford), (2006).
60. DJ Amit, DJ Amit, *Modeling Brain Function: The World of Attractor Neural Networks.* (Cambridge University Press), (1992).
61. G Tkacik, et al., Thermodynamics and signatures of criticality in a network of neurons. *Proc. Natl. Acad. Sci. United States Am.* (2015).
62. D Plenz, et al., Self-Organized Criticality in the Brain. *Front. Phys.* **0** (2021).
63. GF Grosu, et al., The fractal brain: Scale-invariance in structure and dynamics. *Cereb. Cortex* p. bhac363 (2022).
64. WL Shew, H Yang, T Petermann, R Roy, D Plenz, Neuronal Avalanches Imply Maximum Dynamic Range in Cortical Networks at Criticality. *J. Neurosci.* **29**, 15595–15600 (2009).
65. MA Muñoz, R Dickman, A Vespignani, S Zapperi, Avalanche and spreading exponents in systems with absorbing states. *Phys. Rev. E* **59**, 6175–6179 (1999).
66. J Alstott, E Bullmore, D Plenz, Powerlaw: A Python package for analysis of heavy-tailed distributions. *PLoS ONE* **9**, e85777 (2014).
67. AJ Fontenele, et al., Criticality between Cortical States. *Phys. Rev. Lett.* **122**, 208101 (2019).
68. C Zanoci, N Dehghani, M Tegmark, Ensemble inhibition and excitation in the human cortex: An Ising-model analysis with uncertainties. *Phys. Rev. E* **99**, 032408 (2019).
69. R Zeraati, V Buendía, TA Engel, A Levina, Topology-dependent coalescence controls scaling exponents in finite networks. *arXiv preprint arXiv:2211.06296* (2022).
70. Aaron Clauset, CR Shalizi, MEJ Newman, Power-Law Distributions in Empirical Data. *SIAM Rev.* **51**, 661–703 (2009).
71. JM Beggs, Addressing skepticism of the critical brain hypothesis. *Front. Comput. Neurosci.* **16**, 703865 (2022).
72. JM Beggs, *The Cortex and the Critical Point: Understanding the Power of Emergence.* (MIT Press, Cambridge, MA, USA), (2022).

73. J Touboul, A Destexhe, Power-law statistics and universal scaling in the absence of criticality. *Phys. Rev. E* **95**, 012413 (2017).
74. A Destexhe, JD Touboul, Is There Sufficient Evidence for Criticality in Cortical Systems? *eNeuro* **8** (2021).
75. MC Morrell, I Nemenman, A Sederberg, Neural criticality from effective latent variables. *Elife* **12**, RP89337 (2024).
76. N Marshall, et al., Analysis of Power Laws, Shape Collapses, and Neural Complexity: New Techniques and MATLAB Support via the NCC Toolbox. *Front. physiology* **7**, 250 (2016).
